# Identification of DNA-PKcs as a primary resistance factor of salinomycin in osteosarcoma cells

**DOI:** 10.18632/oncotarget.12712

**Published:** 2016-10-17

**Authors:** Yun-fang Zhen, Song-tao Li, Yun-rong Zhu, Xiao-dong Wang, Xiao-zhong Zhou, Lun-qing Zhu

**Affiliations:** ^1^ The Center of Diagnosis and Treatment for Children's Bone Diseases, The Children's Hospital Affiliated to Soochow University, Suzhou, China; ^2^ The Department of Orthopedics, The Second Affiliated Hospital of Soochow University, Suzhou, China; ^3^ Department of Orthopedics, The Affiliated Jiangyin Hospital of Medical College of Southeast University, Jiangyin, China

**Keywords:** osteosarcoma (OS), salinomycin, DNA-PKcs, microRNA-101, autophagy

## Abstract

Malignant osteosarcoma (OS) is still a deadly disease for many affected patients. The search for the novel anti-OS agent is extremely urgent and important. Our previous study has proposed that salinomycin is a novel anti-OS agent. Here we characterized DNA-dependent protein kinase catalytic subunit (DNA-PKcs) as a primary salinomycin resistance factor in OS cells. DNA-PKcs inhibitors (NU7026, NU7441 and LY294002) or DNA-PKcs shRNA knockdown dramatically potentiated salinomycin-induced death and apoptosis of OS cells (U2OS and MG-63 lines). Further, forced-expression of microRNA-101 (“miR-101”) downregulated DNA-PKcs and augmented salinomycin's cytotoxicity against OS cells. Reversely, over-expression of DNA-PKcs in OS cells inhibited salinomycin's lethality. For the mechanism study, we show that DNA-PKcs is required for salinomycin-induced pro-survival autophagy activation. DNA-PKcs inhibition (by NU7441), shRNA knockdown or miR-101 expression inhibited salinomycin-induced Beclin-1 expression and autophagy induction. Meanwhile, knockdown of Beclin-1 by shRNA significantly sensitized salinomycin-induced OS cell lethality. *In vivo*, salinomycin administration suppressed U2OS xenograft tumor growth in severe combined immuno-deficient (SCID) mice, and its anti-tumor activity was dramatically potentiated with co-administration of the DNA-PKcs inhibitor NU7026. Together, these results suggest that DNA-PKcs could be a primary resistance factor of salinomycin in OS cells. DNA-PKcs inhibition or silence may thus significantly increase salinomycin's sensitivity in OS cells.

## INTRODUCTION

Osteosarcoma (OS) is one of leading causes of cancer-related mortalities among children and teenagers [1–4]. Its incidence has been steadily rising in the past decade [1–4]. Although several major improvements have been achieved in diagnosis and treatments for OS, the five-year overall survival for those with malignant OS is still far from satisfactory [1–4]. The malignant OS is resistant to many chemotherapeutic drugs [5]. Therefore, the search for novel ant-OS agents is urgent [1–3].

Recent studies have proposed salinomycin as a novel and efficient anti-cancer agent [6–13]. Our previous study has also demonstrated that salinomycin induced apoptosis and cytotoxicity in human OS cells [11]. Interestingly, we found that salinomycin treatment in OS cells could also induce cytoprotective autophagy activation as downstream of AMPK, which served as a negative regulator against cell apoptosis [11]. Reversely, inhibition of the AMPK-autophagy pathway dramatically potentiated salinomycin's lethality against OS cells [11]. The aim of this study is to investigate the underlying mechanism of salinomycin-induced autophagy activation through focusing on the involvement of DNA-PK catalytic subunit (DNA-PKcs).

DNA-dependent protein kinase (DNA-PK) is a multi-protein complex that is primarily composed of three proteins, including DNA-PKcs and the two Ku hetero-dimer (Ku-70 and Ku-80) [14, 15]. Several other proteins were also found in the complex [14, 15]. DNA-PKcs is 460-kDa serine/threonine protein kinase that belongs to phosphatidylinositol 3-kinase (PI3K)-like protein kinase (PIKK) kinase family [16]. DNA-PKcs will be activated when facing DNA damages, and its normal function is to provoke non-homologous end joining (NHEJ) pathway to repair DNA double strand breaks [14, 15]. Recent studies, however, have proposed oncogenic functions of DNA-PKcs in multiple cancers [17–21]. To our best knowledge, however, the potential role of DNA-PKcs in salinomycin-induced anti-cancer activity has not been studied. We here show that DNA-PKcs could be a primary resistance factor of salinomycin in OS cells. DNA-PKcs inhibition or silence could dramatically sensitize salinomycin's anti-OS activity *in vitro* and *in vivo*.

## RESULTS

### DNA-PK inhibitors dramatically potentiate salinomycin-induced cytotoxicity in OS cells

**Figure 1:**
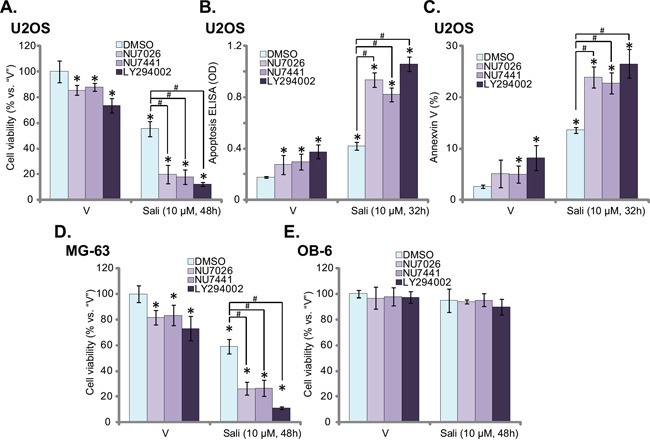
DNA-PK inhibitors dramatically potentiate salinomycin-induced cytotoxicity in OS cells U2OS cells **A-C.**, MG-63 cells **D.** or OB-6 osteoblastic cells **E.** were treated with vehicle (“V”, 0.1 % of DMSO), salinomycin (“Sali”, 10 μM), with/out NU7026 (10 μM), NU7441 (10 μM) or LY294002 (1 μM), cells were further cultured for applied time, and cell survival was tested by CCK-8 assay (A, D and E); Cell apoptosis was tested by histone-DNA ELISA assay (B) or Annexin V FACS assay (C). For each assay, n=5. Experiments in this figure were repeated three times, and similar results were obtained. The data presented were mean ± standard deviation (SD). *p<0.05 *vs.* “V” group. ^#^*p*<0.05 *vs.* “Sali” only group.

In order to test the potential role of DNA-PKcs on salinomycin-induced anti-OS activity *in vitro*, a number of DNA-PKcs inhibitors were applied, including NU7026 [22], NU7441 [23] and LY294002 [24]. In line with our previous findings [11], treatment of U2OS cells with salinomycin (10 μM) induced viability reduction (Figure 1A) and apoptosis activation (Figure 1B and 1C). The latter was tested by the Histone DNA ELISA assay (Figure 1B) and Annexin V FACS assay (Figure 1C) [11]. Significantly, co-treatment with the DNA-PKcs inhibitors (NU7026, NU7441 or LY294002) dramatically sensitized salinomycin's activity, leading to profound cell death (Figure 1A) and apoptosis (Figure 1B and 1C). The similar results were also observed in MG-63 OS cells, where co-treatment of the DNA-PK inhibitors dramatically augmented salinomycin's lethality (Figure 1D). Intriguingly, the DNA-PKcs inhibitors alone also induced mild cytotoxicity to the OS cells (Figure 1A-1D). Notably, same salinomycin plus DNA-PKcs inhibitor treatment failed to induce significant cytotoxicity to the non-cancerous OB-6 osteoblastic cells, suggesting cancer cell specific response by the co-treatment (Figure 1E). Together, these *in vitro* results show that DNA-PKcs inhibitors dramatically sensitize salinomycin-induced cytotoxicity against human OS cells.

### Salinomycin's sensitivity against OS cells is increased with DNA-PKcs knockdown, but decreased with DNA-PKcs over-expression

**Figure 2:**
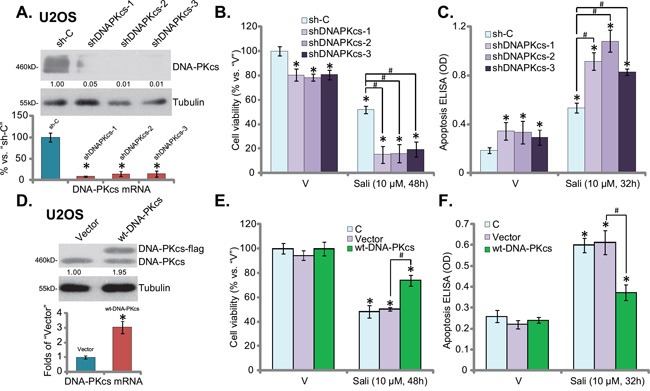
Salinomycin's sensitivity in OS cells is increased with DNA-PKcs knockdown, but decreased with DNA-PKcs over-expression Stable U2OS cells, expressing scramble control shRNA (“sh-C”) or three different DNA-PKcs-shRNA (“shDNAPKcs-1/2/3”, see Method) as well as wt-DNA-PKcs or the empty vector (“pSV2-neo”), were treated with vehicle (“V”, 0.1 % of DMSO) or salinomycin (“Sali”, 10 μM) for applied time, DNA-PKcs protein/mRNA expression **A** and **D.**, cell survival **B** and **E.**, CCK-8 assay) and cell apoptosis **C** and **F.**, Histone DNA ELISA assay) were tested by listed assays. DNA-PKcs protein expression (*vs.* Tubulin) was quantified (A and D, upper panels). “C” stands for un-transfected control cells (E and F). For each assay, n=5. Experiments in this figure were repeated three times, and similar results were obtained. The data presented were mean ± standard deviation (SD). *p<0.05 *vs.* “V” group. ^#^*p*<0.05.

Above results showed that DNA-PKcs inhibitors potentiated salinomycin-induced lethality against OS cells. To rule out the possible off-target effect of the DNA-PKcs inhibitors, in particularly, LY294002 is also a PI3K-Akt-mTOR pan inhibitor [25], we next utilized genetic strategies to change DNA-PKcs expression. First, three different lentiviral shRNAs, targeting non-overlapping sequences of DNA-PKcs mRNA (see Methods), were applied. All of them efficiently and specifically downregulated DNA-PKcs protein and mRNA expression in U2OS cells (Figure 2A). Importantly, salinomycin-induced viability reduction and apoptosis were significantly augmented in DNA-PKcs-silenced U2OS cells, suggesting again that DNA-PKcs could be a primary resistance factor of salinomycin. Notably, U2OS cells with DNA-PKcs shRNA also presented with moderately reduced cell survival (Figure 2B), but slightly increased cell apoptosis (Figure 2C), as compared to the control cells. Thus, basal DNA-PKcs expression is important for U2OS cell survival.

Based on the results above, we would speculate that DNA-PKcs over-expression may inhibitsalinomycin's cytotoxicity in OS cells. Therefore, a wt-DNA-PKcs expression vector was introduced to cultured U2OS cells. As shown in Figure 2D, DNA-PKcs protein (upper panel) and mRNA (lower panel) expression was indeed significantly increased after transfection. Consequently, salinomycin-induced cell death (Figure 2E) and apoptosis (Figure 2F) were largely attenuated in DNA-PKcs-over-expressed U2OS cells. Notably, we repeated the shRNA and over-expression experiments in MG-63 cells, and similar results were achieved (data not shown).

### miR-101 downregulates DNA-PKcs and augments salinomycin's cytotoxicity in OS cells

**Figure 3:**
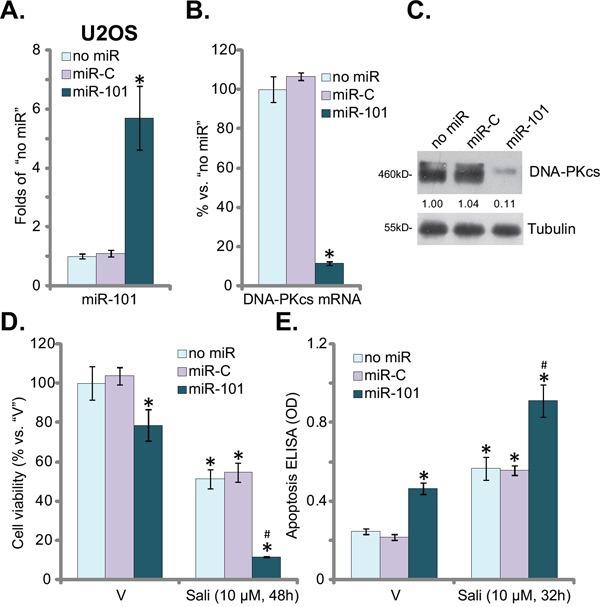
miR-101 downregulates DNA-PKcs and augments salinomycin's cytotoxicity in OS cells Stable U2OS cells, expressing microRNA-101 (“miR-101”), miRNA control (“miR-C”) or the control U2OS cells (“no miR”) were subjected to real-time PCR assay, miR-101 expression **A.** and DNA-PKcs mRNA expression **B.** were shown; DNA-PKcs protein expression was also tested **C.**; Above cells were treated with vehicle (“V”, 0.1 % of DMSO) or salinomycin (“Sali”, 10 μM) for applied time, cell viability **D.**, CCK-8 assay and apoptosis **E.**, Histone DNA ELISA assay) were tested. DNA-PKcs protein expression (*vs.* Tubulin) was quantified (C). For each assay, n=5. Experiments in this figure were repeated three times, and similar results were obtained. The data presented were mean ± standard deviation (SD). *p<0.05 *vs.* “V” group. ^#^ *vs.* “miR-C” group.

Recent studies have demonstrated that microRNA-101 (“miR-101”) is anti-DNA-PKcs microRNA [26, 27]. In the current study, miR-101-exprsssion vector (a gift from Dr. Lu [28]) was introduced to U2OS cells, and stable cells were selected. Real-time quantitative PCR (“RT-qPCR”) assay results confirmed miR-101 over-expression in the stable cells (Figure 3A). Consequently, DNA-PKcs mRNA (Figure 3B) and protein (Figure 3C) expression was significantly downregulated. These results again imply that miR-101 selectively targets and downregulates DNA-PKcs in OS cells.

Significantly, U2OS cells with miR-101 over-expression became vulnerable to salinomycin, which induced profound cytotoxicity (Figure 3D) and apoptosis (Figure 3F) in these cells. Notably, miR-101 expression alone also induced moderate cell death (Figure 3D) and apoptosis (Figure 3E) in U2OS cells, which are in line with the DNA-PKcs inhibitor (Figure 1) and shRNA data (Figure 2). As expected, miRNA-control (“miR-C”) didn't affect DNA-PKcs expression (Figure 3B and 3C) or salinomycin's sensitivity (Figure 3D and 3E). We repeated the miR-101 experiments in MG-63 cells, and similar results were obtained (Data not shown). Collectively, these results suggest that miR-101 downregulates DNA-PKcs and potentiates salinomycin's cytotoxicity in OS cells.

### DNA-PKcs is required for salinomycin-induced autophagy activation in OS cells

**Figure 4:**
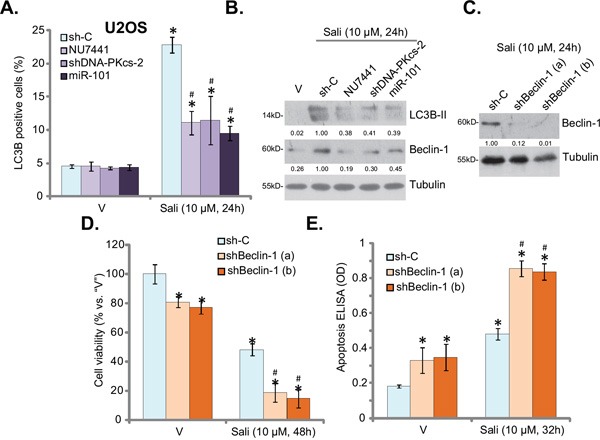
DNA-PKcs is required for salinomycin-induced autophagy activation in OS cells Stable U2OS cells, expressing scramble control shRNA (“sh-C”), DNA-PKcs-shRNA (“shDNAPKcs-2”) or microRNA-101 (“miR-101”), were treated with vehicle (“V”, 0.1 % of DMSO), salinomycin (“Sali”, 10 μM) or plus NU7441 (10 μM) for indicated time, the percentage of LC3B puncta positive cells was shown **A.**; The expression of listed proteins was detected by Western blot assay **B**. Stable U2OS cells, expressing scramble control shRNA (“sh-C”) or Beclin-1-shRNA (“shBeclin-1 a/b”) were treated with vehicle (“V”, 0.1 % of DMSO) or salinomycin (“Sali”, 10 μM) for applied time, Beclin-1 expression **C**., cell survival CCK-8 assay, **D**. and apoptosis histone DNA ELISA assay, **E**. were tested. LC3B-II, Beclin-1 protein expressions (*vs.* Tubulin) were quantified (B and C). For each assay, n=5. Experiments in this figure were repeated three times, and similar results were obtained. The data presented were mean ± standard deviation (SD). *p<0.05 *vs.* “V” group. ^#^*p*<0.05 *vs.* “Sali” only group.

Our previous study has shown that salinomycin activated cyto-protective autophagy in OS cells, which functioned as a negative regulator against cell apoptosis [11]. We thus wanted to know whether DNA-PKcs played a role in salinomycin-induced autophagy. In line with our previous findings [11], salinomycin treatment in U2OS cells increased LC3B puncta formation (Figure 4A) and the autophagy marker LC3B-II expression (Figure 4B), indicating autophagy activation. Significantly, DNA-PKcs inhibition (by NU7441), shRNA knockdown, or miR-101 expression largely inhibited autophagy activation by salinomycin in USO2 cells (Figure 4A-4C). Based on these results, we suggest that salinomycin-induced autophagy activation requires DNA-PKcs in OS cells.

For the mechanism study, we tested the potential role of DNA-PKcs in regulating autophagy-associated proteins. Induction of autophagy is usually accompanied with increase of microtubule-associated protein Beclin-1 [29]. We found that salinomycin also increased Beclin-1 expression in U2OS cells (Figure 4B). Remarkably, shRNA-mediated knockdown of Beclin-1 (Figure 4C) dramatically enhanced salinomycin-induced cytotoxicity in U2OS cells (Figure 4D and 4E). More importantly, inhibition or knockdown of DNA-PKcs by the above means also inhibited salinomycin-induced Beclin-1 expression in U2OS cells (Figure 4B), suggesting that DNA-PKcs is required for salinomycin-induced Beclin-1 expression. This could be the key mechanism of DNA-PKcs-mediated resistance against salinomycin in OS cells. Notably, Beclin-1 shRNA knockdown alone also induced minor U2OS cell death and apoptosis (Figure 4D and 4E), suggesting that basal autophagy activation is important for USO2 cell survival.

### NU7026 potentiates salinomycin-induced anti-tumor activity *in vivo*

**Figure 5:**
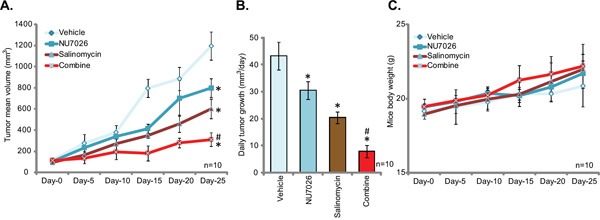
NU7026 sensitizes salinomycin-induced anti-tumor activity *in vivo* U2OS-bearing SCID mice were administrated with saline (“Vehicle”), salinomycin (5 mg/kg, daily, gavage) and/or NU7026 (50 mg/kg, daily, IP), tumor volumes **A.** and mice body weights **C.** were recorded every five days; Daily tumor growth was also calculated **B.** Experiments in this figure were repeated twice, and similar results were obtained. The data presented were mean ± standard deviation (SD). *p<0.05 *vs.* “Vehicle” group. ^#^ *p*<0.05 *vs.* “Salinomycin” only group.

At last, we tested the potential anti-OS activity of salinomycin *in vivo*, using a U2OS xenograft SCID mice model [30]. As described previously [30], a significant number of U2OS cells were inoculated into the SCID mice, and within 2-3 weeks the xenograft tumors were established. As demonstrated, oral administration of salinomycin at 5 mg/kg (daily, gavage) inhibited U2OS xenograft growth in SCID mice (Figure 5A), confirming its anti-OS activity *in vivo*. Remarkably, co-administration with NU7026 (50 mg/kg, daily, IP) significantly enhanced salinomycin-induced anti-tumor activity, and U2OS tumor growth in mice with the co-administration was dramatically inhibited (Figure 5A). NU7026 alone also slightly inhibited U2OS tumor growth (Figure 5A). The anti-tumor activity by the combination was apparently more potent than either single treatment (Figure 5A). Daily tumor growth results in Figure 5B further showed the superior anti-tumor activity by the combo. Salinomycin plus NU7026 co-administration led to over 75% of inhibition of daily tumor growth, as compared to the vehicle control (Figure 5B). Interestingly, the mice body weights were not significantly affected by the single or the combination treatment (Figure 5C). These mice were thus tolerate to the treatment regimens. These results demonstrate that DNA-PKcs inhibition by NU7026 sensitizes salinomycin-induced anti-tumor activity *in vivo.*

## DISCUSSION

Recent studies have been focusing on expression and biological functions of DNA-PKcs in several human cancers [17, 19, 20, 31–34]. The results have proposed that DNA-PKcs could be an important oncogene which promotes cancer initiation, progression, and apoptosis-resistance [17, 19, 20, 31–35]. In many different cancers, DNA-PKcs is over-expressed and represents a novel and important oncotarget [17, 19, 20, 31–35].

The results of the current study suggest that DNA-PKcs is primary resistance factor of salinomycin at least in OS cells. First, DNA-PKcs inhibition or shRNA knockdown dramatically potentiated salinomycin-induced OS cell death and apoptosis; Second, expression of miR-101, an anti-DNA-PKcs miRNA [26, 36], downregulated DNA-PKcs and augmented salinomycin's lethality in OS cells; Third, forced-expression of DNA-PKcs in OS cells inhibited salinomycin's cytotoxicity; Fourth, salinomycin-mediated anti-tumor activity *in vivo* was dramatically sensitized with co-administration of DNA-PKcs inhibitor NU7026. Based on these results, we conclude that salinomycin-induced anti-OS activity should be significantly sensitized with DNA-PKcs inhibitor or silence. It would be interesting to test this scenario in other cancer cells.

It should be noted that salinomycin or plus DNA-PKcs inhibitors failed to induce significant death of non-cancerous OB-6 osteoblastic cells. This could be due to salinomycin's selective cytotoxicity only to cancerous cells, as shown by many other studies [8, 9, 12, 13, 37]. Another possibility is that DNA-PKcs expression level is indeed quite low in OB-6 cells (Data not shown), as compared to the OS cells studied here.

There are several explanations for DNA-PKcs-mediated oncogenic actions. For example, it has been shown that DNA-PKcs over-expression in many cancer cells mediates activation of Akt-mTOR signaling, the latter is a major pro-survival and chemo-resistance signaling [38, 39]. DNA-PKcs could form a complex with Akt, leading to ten-fold increase of Akt activity [40]. Another reason could be due to DNA-PKcs's ability to repair damaged DNA. DNA damages are often observed in OS cells and many cancer cells, serving as an important apoptosis resistance factor [41–43]. DNA-PKcs inhibition, mutation or depletion therefore may disrupt DNA repair process, and favor a pro-apoptosis outcome [41–43]. As a matter of fact, in this study, we showed that DNA-PKcs inhibition or silence also moderately induced OS cell death and apoptosis.

Interestingly, here we propose that DNA-PKcs is required for salinomycin-induced autophagy activation, which is pro-survival in OS cells (See other previous study [11]). DNA-PKcs inhibition, shRNA knockdown or miR-101 expression inhibited salinomycin-induced Beclin-1 expression and autophagy induction. This could be the primary mechanism of DNA-PKcs-mediated resistance against salinomycin. To support this hypothesis, we show that shRNA-mediated Beclin-1 also significantly potentiated salinomycin-mediated lethality in OS cells.

In summary, these results suggest that DNA-PKcs could be a primary resistance factor of salinomycin in OS cells. Inhibition or silence of DNA-PKcs could significantly increase salinomycin's sensitivity in OS cells.

## MATERIALS AND METHODS

### Chemicals and reagents

Salinomycin was obtained from Sigma (Sigma, St. Louis, MO). DNA-PKcs inhibitors LY294002, NU-7026 and NU-7441 were purchased from Calbiochem (Shanghai, China). All the antibodies utilized in this study were purchased from Cell Signaling Tech (Denver MA). The enhanced chemiluminescence (ECL) reagent kit was purchased from Pierce (Rockford, IL). All cell culture reagents were purchased from Gibco BRL (Shanghai, China).

### Cell culture

U2OS and MG-63 human OS cells were maintained in DMEM plus 10% FBS and penicillin/streptomycin (1:100), in a CO_2_ incubator at 37°C [11]. The non-cancerous OB-6 human osteoblastic cells [44] were purchased from the Cell Bank of Shanghai Institute of Biological Science (Shanghai, China), and were maintained as described [44].

### CCK-8 cell viability assay

Following treatment of cells, the viability was measured by Cell Counting Kit-8 (CCK-8) (Dojindo, Japan) assay according to manufacturer's protocol. The OD value of the treatment group was normalized to that vehicle control group [11]. Cell viability reduction was detected as the indicator of cell death [11].

### Analysis cell apoptosis by flow cytometry assay

As previously described [11], after indicated treatment, cell apoptosis was detected via the Annexin V Apoptosis Detection Kit (Biyuntian, Shanghai, China) according to the manufacturer's protocol. Both early (Annexin V^+^/PI^−^) and late (Annexin V/PI^+^) apoptotic cells were gated by fluorescence-activated cell sorting (FACS) (Beckman Coulter, Suzhou, China). Annexin V percentage ratio was recorded as the quantitative indicator of cell apoptosis.

### Cell apoptosis detection by enzyme-linked immunosorbent assay (ELISA)

As described in our previous studies [11, 45, 46], after indicated treatments, the Histone-DNA ELISA Detection Kit (Roche, Palo Alto, CA) was utilized to quantify cell apoptosis via ELISA method, according to the manufacturer's protocol.

### Western blot

Cells with applied treatment were incubated in the lysis buffer as described [11]. The protein lysates (30 μg/sample) were separated by 10% SDS-polyacrylamide gel, and electro-transferred onto a polyvinylidene fluoride (PVDF) membrane (Millipore, USA). Afterwards, the membrane was blocked, followed by incubation with specific primary and secondary antibodies. The detection of indicated protein was performed by ECL Supersingnal West Pico Chemiluminescent. The total gray of indicated band was quantified via ImageJ software, and was normalized to the loading control.

### Real-time quantitative PCR assay

The protocol of real-time quantitative reverse transcriptase polymerase chain reaction (“RT-qPCR”) assay was described in detail in our previous study [11]. The comparative Ct (2^−ΔΔCt^) method was applied to calculate relative mRNA expression level [47]. Glyceraldehyde-3-phosphate dehydrogenase (GAPDH) was tested as the reference gene [11]. The expression of mature microRNA-101 (“miR-101”) was tested by the TaqMan microRNA assay as described [48]. Ten ng of total RNA per sample was reverse-transcribed via the TaqMan MicroRNA Reverse Transcription Kit (Applied Biosystem, Shanghai, China) [48]. The primer sequences were as follows: DNA-PKcs primers, forward 5′-CCAAGTCCAACACCAAGTAGCCACCCA-3′; and reverse 5′-CCGCCATGCCGCCGAGTCCC-3′ [43]. GAPDH primers, forward, 5′-GAAGGTGAAGGTCGGAGTC-3′; reverse, 5′-GAAGATGGTGATGGGATTTC-3′; miR-101: forward: 5′- CGG CGG TAC AGT ACT GTG ATA A-3′, reverse: 5′- CTG GTG TCG TGG AGT CGG CAA TTC-3′ (Universal stem-loop primer) [28, 49]. All the primers were synthesized by Genepharm (Shanghai, China).

### shRNA knock and stable cell selection

The two different lentiviral shRNAs (GV248-puromycin vector, “-1/−2”) against human DNA-PKcs were gifts from Dr. Bing Zheng [27]. The third DNA-PKcs shRNA (“-3”) was purchased from Santa Cruz Biotech (sc-35200-V). The two different lentiviral Beclin-1 shRNAs were purchased from Santa Cruz Biotech (sc-29797-V, “Beclin-1 shRNA-a”) and Genepharm (Shanghai, China, “Beclin-1 shRNA-b”), respectively. The scramble control shRNA (sc-108065) were also purchased from Santa Cruz Biotech. For shRNA experiment, OS cells were seeded onto six-well plates with 60% confluence. Ten μL/mL of lentiviral shRNA was added to cultured OS cells for 36 hours. Afterwards, cells were subjected to puromycin (1 μg/mL, Sigma) selection for another 48 hours. The knockdown of DNA-PKcs or Beclin-1 in the stable cells was verified by Western blot and/or RT-qPCR assay.

### LC3B immunochemistry

As described previously [11], following the treatment, cells were fixed, washed and blocked. The slides were then incubated with the primary antibody (anti-LC3B, Cell Signaling Tech, 1:25) and FITC-conjugated second antibody (Biyuntian). Afterwards, LC3B fluorescence was visualized via a Leica microscope. The percentage LC3B puncta positive cells (green fluorescence) was recorded. For each count, a total of at least 200 cells (TUNEL stained) in each view from independent treatment were counted [11].

### DNA-PKcs overexpression

The wild-type (wt-) DNA-PKcs pSV2-neo-Flag plasmid is a gift from Dr. Lu at Nanjing Medical University [36]. The construct was transfected into U2OS cells via Lipofectamine 2000 (Invitrogen) [36]. After 36 hours, U2OS cells were re-plated on selection medium with 100 μg/mL of G418 for 48 hours. Expression of DNA-PKcs (Flag-tagged) in the stable cells was again tested by Western blot assay and/or RT-qPCR assay.

### miRNA construct and transfection

The miR-101 pSuper-puro-GFP vector and miR-control (“miR-C”) vector were gifts from Dr. Lu [28]. Cells were transfected with miR-101 or miR-C vector using Lipofectamine 2000 transfection reagent. After 36 hours, cells were subjected to puromycin (1 μg/mL) selection for another 48 hours. Expression of miR-101 in the stable cells was always tested by RT-qPCR assay.

### Mice U2OS xenograft assay

As described previously [30, 50], CB.17 severe combined immuno-deficient (SCID) male mice (18-20g, purchased from Soochow University Animal Facility, Suzhou, China) were applied for *in vivo* xenograft experiments. Three million U2OS cells per mouse were injected subcutaneously (*s.c.*) into the right flanks of the mice. After about three weeks when the xenografts were about 100 mm^3^ in volume, and the SCID mice (10 mice per group) were randomly divided into four groups: Vehicle control (Saline), salinomycin (5 mg/kg, gavage [51]), NU7026 (50 mg/kg, intraperitoneal injection, IP) [22] or salinomycin plus NU7026 co-administration. The agents were freshly prepared and given daily for a total of 20 days. The xenografted tumor diameter was measured every 5 days. Tumor volumes (mm^3^) and mice body weights (g) were recorded as described [30, 50, 52]. The protocols were in accordance with the Institutional Animal Care and Use Committee (IACUC), and were approved by the Ethics Committee and Internal Review Board (IRB) of all authors' institutions.

### Statistical analysis

The quantitative data presented in this study was mean ± standard deviation (SD). Statistical differences were analyzed by one-way ANOVA with post hoc Bonferroni test (SPSS version 18.0). Values of ***p***<0.05 were considered statistically different.
